# Preventing Breast, Cervical, and Colorectal Cancer Deaths: Assessing the Impact of Increased Screening

**DOI:** 10.5888/pcd17.200039

**Published:** 2020-10-08

**Authors:** Krishna P. Sharma, Scott D. Grosse, Michael V. Maciosek, Djenaba Joseph, Kakoli Roy, Lisa C. Richardson, Harold Jaffe

**Affiliations:** 1 Division of Cancer Prevention and Control, National Center for Chronic Disease Prevention and Health Promotion, Centers for Disease Control and Prevention, Atlanta, Georgia; 2 National Center on Birth Defects and Developmental Disabilities, Centers for Disease Control and Prevention, Atlanta, Georgia; 3 HealthPartners Institute, Minneapolis, Minnesota; 4 National Center for Chronic Disease Prevention and Health Promotion, Centers for Disease Control and Prevention, Atlanta, Georgia; 5 Office of the Associate Director for Science, Centers for Disease Control and Prevention, Atlanta, Georgia

## Abstract

**Introduction:**

The US Preventive Services Task Force (USPSTF) recommends select preventive clinical services, including cancer screening. However, screening for cancers remains underutilized in the United States. The Centers for Disease Control and Prevention leads initiatives to increase breast, cervical, and colorectal cancer (CRC) screening. We assessed the number of avoidable deaths from increased screening, according to USPSTF recommendations, for CRC and female breast and cervical cancers.

**Methods:**

We used model-based estimates of avoidable deaths for the lifetime of single-year age cohorts under the current and increased use of screening scenarios (data year 2016; analysis, 2018). We calculated prevented cancer deaths for each 1% increase in screening uptake and extrapolated to current level of screening (2016), current level plus 10 percentage points, and increasing screening to 90% and 100% of the eligible population.

**Results:**

Increased use of screening from current levels to 100% would prevent an additional 2,821 deaths from breast cancer, 6,834 deaths from cervical cancer, and 35,530 deaths from CRC over a lifetime of the respective single-year cohort. Increasing use of CRC screening would prevent approximately 8.5 times as many deaths as the equivalent increase in use of breast cancer screening (women only), although twice as many people (men and women) would have to be screened for CRC.

**Conclusions:**

A large number of deaths could be avoided by increasing breast, cervical, and CRC screening. Public health programs incorporating strategies shown to be effective can help increase screening rates.

**Summary**

**What is already known on this topic?**

Screening for colorectal cancer and for female breast and cervical cancers can effectively reduce deaths from these cancers. Yet many preventive services, including cancer screening, remain underutilized in the United States.

**What is added by this report?**

Increased use of screening from current levels to 100% would prevent an additional 2,821 deaths from breast cancer, 6,834 deaths from cervical cancer, and 35,530 deaths from colorectal cancer over a lifetime of the respective single-year cohort. Increasing use of colorectal cancer screening would prevent more deaths than an equivalent increase in breast and cervical cancer screening.

**What are the implications for public health practice?**

Public health programs incorporating strategies shown to be effective can help increase screening rates. Organized screening approaches leveraging partnerships between public health and primary health care to implement such strategies could be used to reduce the prevalence of these cancers.

## Introduction

The US Preventive Services Task Force (USPSTF) recommends select clinical preventive services with “A” and “B” recommendation grades for the eligible population. A grade “A” recommendation reflects high certainty of substantial net benefit from a service; grade “B” reflects high certainty of moderate benefit or moderate certainty of substantial benefit. USPSTF recommendations include routine screening for female breast cancer in women aged 50 to 74 years, cervical cancer in women aged 21 to 65 years, and colorectal cancer (CRC) in men and women aged 50 to 75 years (1). Most private health plans cover these services without copays or deductibles. However, insurance coverage does not ensure uptake of recommended services, and many preventive services remain underutilized (2).

To increase the use of these services, the US Department of Health and Human Services supports various programs and initiatives (3). For example, 2 cancer control programs at the Centers for Disease Control and Prevention (CDC), the National Breast and Cervical Cancer Early Detection Program (NBCCEDP) and the Colorectal Cancer Control Program (CRCCP), seek to increase screening use among low-income, medically underserved populations (4,5). Despite the availability of screening services and better treatment outcomes, a large number of patients still die of these cancers. In 2016, the number of deaths from female breast cancer was 41,487; from cervical cancer, 4,188; and from CRC, 52,286 (6). In 2016, the self-reported screening rates for female breast and cervical cancers were 78.3% and 79.9%, respectively, and the self-reported screening rate for CRC was 67.7% (7). 

In this article, we assess the number of potential deaths that could be prevented by increasing screening for female breast and cervical cancers and for CRC according to USPSTF recommendations. The report is motivated by the need to increase the use of evidence-based interventions that reduce the rates of illness and death from cancer.

## Methods

We simulated and compared the number of deaths that could be prevented by increasing screening from current rates to defined targets by using previously reported model-based estimates. We compared the cumulative numbers of cancer deaths for a single-year age cohort under different scenarios: current level of screening (2016), current level plus 10 percentage points, and increasing screening to 90% and 100% of the eligible population. We also calculated the numbers of adults currently screened and expected to be screened under different scenarios of increased screening. Table 1 provides a summary of key analysis assumptions and model inputs. Current screening estimates are based on 2016 survey data from the Behavioral Risk Factor Surveillance System (BRFSS) (7).

**Table 1:** Summary of Key Analysis Assumptions Used to Estimate the Effects of Colorectal Cancer and Women’s Breast and Cervical Cancers in the United States

Analysis Assumption	Breast Cancer	Cervical Cancer	Colorectal Cancer
Study cohort	50-year-old women	21-year-old women	50-year-old men and women
Screening age^a^	50–74 y	21–65 y	50–75 y
Eligible US population for the test (million)^b^	48.7	96.7	95.9
Follow-up period	Lifetime or until death by any cause		
Screening tests included^a^	Mammogram	Cytology or pap smear	High-sensitivity FOBT, flexible sigmoidoscopy, or colonoscopy
Screening intervals^a^	Every 2 years	Every 3 years	Annual screening with high-sensitivity FOBT Sigmoidoscopy every 5 years, with high-sensitivity FOBT every 3 years Screening colonoscopy every 10 years
Baseline screening rate (%)^c^	78.3	79.9	67.7
Age eligible US population screened in baseline (millions)^b^	37.4	76.8	63.5
Other screening scenarios (number of additional people needed to be screened to reach the goal [in millions] by cancer type^b^)	Increase in baseline rate by 10 percentage points (breast, 4.8; cervical, 9.6; colorectal, 9.4) Screening rate of 90% (breast, 5.6; cervical, 9.7; colorectal, 20.9) Screening rate of 100% (breast, 10.4; cervical, 19.3; colorectal, 30.3)		

Abbreviations: BRFSS, Behavioral Risk Factor Surveillance System; FOBT, fecal occult blood test.

a Based on US Preventive Services Task Force recommendations, 2008.

b Author calculations based on annual estimates of the resident population by sex, race, and Hispanic origin for 2016 from the US Census.

c Based on BRFSS 2016 data (7).

Each of the simulation models on which our calculations are based followed a synthetic cohort from the USPSTF-recommended starting age of screening: 50-year-old women for breast cancer screening, 21-year-old women for cervical cancer screening, and 50-year-old men and women for CRC screening. The simulations followed each cohort through their lifetimes. Screening modalities included mammography for breast cancer and cytology or Pap test for cervical cancer. For CRC, the model assumed a mix of annual fecal occult blood test (FOBT), flexible sigmoidoscopy every 5 years plus FOBT every 3 years, or colonoscopy every 10 years (Table 1).

The estimates of avoidable burden were prepared in 2018 by Health Partners Institute researchers using models that were previously used in peer-reviewed studies to inform the National Commission on Prevention Priorities (NCPP) ranking of clinical preventive services (8). Specifically, the estimates for avoidable deaths from breast cancer screening (9) were based on results of 5 Cancer Information Surveillance Modeling Network screening models (10) plus an estimate from a sixth model (11). Estimates for cervical cancer screening and CRC screening were based on results from models to inform the same NCPP ranking (12,13). These reports provide estimates of cancer deaths that would be prevented either by screening 100% of the target population compared with no screening (8,9) or by screening a portion of the target population who would accept and follow up with screening if recommended by a physician (10,11,14). Each model estimated cancer deaths prevented by first constructing a natural history of cancer based on progression of lesions through cancer stages and then simulating the potential for screening to interrupt cancer progression and prevent death. Using the estimates from models, we calculated the deaths prevented from each 1% increase in screening uptake in the US eligible population and linearly scaled that estimate from current screening rates up to the screening rates in the scenarios just described. Linear extrapolation should provide a reasonable estimate of the impact of increasing screening rates when capacity exists or is developed to provide additional screening and follow-up of quality equal to existing screening and follow-up, and when the currently screened and unscreened populations have similar risks of lesion development and cancer progression.

## Results

If the current level of screening use were maintained, 10,179 deaths from breast cancer would be prevented among the cohort of 50-year-old women over their lifetime; 27,166 deaths from cervical cancer would be prevented among the cohort of 21-year-old women; and 74,470 deaths from CRC would be prevented among the cohort of 50-year-old men and women (Table 2).

**Table 2:** Estimates of Current and Increased Use of US Preventive Services Task Force–Recommended Cancer Screenings Over the Lifetime of Study Cohort, United States, 2018

Preventive Service	Current Use, %^a^	Current Impact (Deaths Prevented)^b^	Incremental Impact (Deaths Prevented) With Increased Screening	
			Increase Screening by 10 Percentage Points^b^	Increase Screening to 90%^b^
Breast cancer screening of 50-year-old women until the age of 74	78.3	10,179	1,300	1,521
Cervical cancer screening of 21-year-old women until the age of 65	79.9	27,166	3,400	3,434
Colorectal cancer screening of 50-year-old adults until the age of 75	67.7	74,470	11,000	24,530

a Source: Behavioral Risk Factor Surveillance System Prevalence and Trends Data (7).

b Model-based estimates by authors.

Using a linear relation between screening use and avoided deaths indicated a similar pattern of relative incremental deaths prevented through increased screening. Increases of 10 percentage points would prevent an additional 1,300 deaths from breast cancer; 3,400 deaths from cervical cancer; and 11,000 deaths from CRC over the lifetime of each cohort. In terms of the 2016 general population, those reductions would require additional screenings of 4.9 million women for breast cancer, 9.7 million women for cervical cancer, and 9.6 million men and women for CRC (Table 1).

The impact of increasing the screening rate to 100% sets the upper limit on the number of potentially avoidable deaths (Figure). Screening of 100% age-appropriate adults could prevent 2,821 additional deaths from breast cancer over the lifetime of a cohort of 50-year-old women; 6,834 additional deaths from cervical cancer over the lifetime of 21-year-old women; and 35,530 additional deaths from CRC over the lifetime of 50-year-old men and women. Increasing use of CRC screening would prevent approximately 8.5 times as many deaths as the equivalent increase in use of breast cancer screening (women only), although twice as many people (men and women) would have to be screened for CRC (Table 1).

**Figure:**
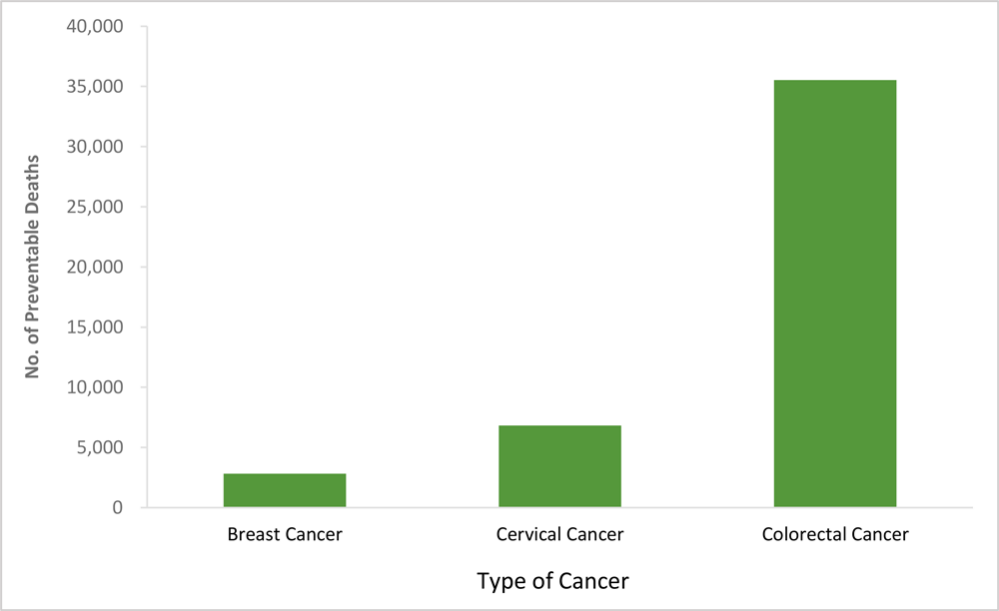
Estimates of maximum number of preventable deaths in a single-year cohort with increased use of screening under US Preventive Services Task Force guidelines (study year 2018). Preventable deaths over a lifetime for breast cancer are among women aged 50, for cervical cancer among women aged 21, and for colorectal cancer among men and women aged 50.

**Table:** 

Cancer Type	No. of Preventable Deaths Over Lifetime
Breast	2,821
Cervical	6,834
Colorectal	35,530

## Discussion

The estimated deaths from breast cancer, cervical cancer, and CRC prevented under different scenarios, comparing the impact of incremental screening rates, may be useful for setting goals and making resource allocation decisions on prevention. For example, one of the goals of Healthy People 2020, the US government’s 10-year national health objectives, is to reduce female breast and cervical cancer mortality by 10% and CRC mortality by 15% (15). Our estimates suggest that large numbers of deaths from cancer could be prevented through increased use of evidence-based screenings. The greatest impact could be realized for increased CRC screening. The magnitude of potential impact of universal CRC screening is attributed to the fact that CRC screening has a current rate that is lower than breast and cervical cancer screening, includes both men and women, and has a larger proportionate decrease in mortality associated with it. Although we recognize that 100% screening is not an achievable goal, we included it as a target to illustrate the maximum benefit that could be achieved by increased screening.

The Community Preventive Services Task Force (CPSTF) recommends evidence-based strategies, such as patient and provider reminders, to increase screening rates for all 3 cancers (16–18). CDC’s CRCCP aims to increase screening rates among priority populations through implementation of these strategies in health system clinic settings. The NBCCEDP is a long-standing CDC initiative that screened over 1.4 million low-income, uninsured and underinsured women over the 5 years ending in 2017 alone (19). These public health programs, along with other state and local efforts, are critical to increasing cancer screening. For example, early results of CRCCP suggested a 4.4 percentage-point annual increase in screening rates among the participating clinics (5). By the second and third year of the CRCCP, the rate increased by 8.3 and 10.1 percentage points, respectively. An increase of 10.1 percentage points implied more than 82,000 additional CRC screenings under CRCCP (20).

Increasing cancer screening rates would require additional resources for the delivery of clinical services, as well as strategies to promote uptake of screening in population groups with lower use of screening. Previous studies that examined the cost of public provision of programs to increase screening found that such programs include not only cost of screening services but also substantial cost of administering and promoting the programs (21,22). The incremental costs associated with additional screenings may be offset by early detection of cancer or precancerous abnormalities through routine screening. In particular, use of colonoscopy for CRC screening or as follow-up to abnormal fecal screening can significantly reduce the onset of CRC through removal of precancerous polyps in addition to allowing early detection of tumors. Consequently, economic analyses have concluded that screening for CRC might be cost-saving to health care systems, with the magnitude of cost savings greater for colonoscopy-based screening (23,24). A CPSTF systematic review found that multicomponent interventions to promote CRC might also be cost-saving, a finding that was based on a small study in a disadvantaged population in south Texas and a modeling study from South Korea (25). However, those analyses did not factor in competing risks or future medical costs, although taking those into account may still render CRC screening to be considered cost-effective even if not cost-saving (26).

Our estimates of the relative contributions of recommended screenings align with previous estimates, although methods differ (27). In particular, the results of Farley et al reflect annual impact in a US cross-section, while our estimates reflect the lifetimes of a US birth cohort. These different methods could produce the same number of life-years at risk of cancer and the same results if, among other things, the successive birth cohorts represented in the cross-section were all the same size. However, because the older cohorts in a cross-section came from smaller, pre-1946 birth cohorts, annual estimates tend to be smaller than lifetime estimates from a birth cohort. Our estimate of 68% (35,530) CRC deaths prevented, associated with increasing screening from 68% to 100%, is higher than the Meester et al estimate (28) of 58% CRC deaths prevented in 2020, even with an increase in screening rate from 60% to 100%.

### Limitations

The current rates of screening used in this study were based on self-reported BRFSS survey data, but actual rates could be substantially less. Past studies have suggested that self-reports of screening overestimated screening rates by as much as 15 to 25 percentage points (29,30). We did not account for the potential contribution from the use of human papillomavirus (HPV) vaccination to reduce incidence of cervical cancer; neither did we include HPV testing for cervical cancer screening in women aged 30 or older. The estimates for CRC deaths prevented were based on 3 screening strategies: FOBT alone, flexible sigmoidoscopy combined with FOBT, and colonoscopy alone; other currently available or recommended strategies or test methods (eg, fecal immunochemical test, fecal DNA, Cologuard) were not included. Furthermore, our approach assumes proportional effects of screening and does not account for population heterogeneity in screening frequencies and risk of death. Also, the validity of our approach to extrapolate outside the observed range of data is not known, although this is often the only approach available.

### Conclusions

Increasing screening for CRC and breast and cervical cancers could prevent a substantial number of deaths attributed to these cancers. Organized screening approaches that leverage partnerships between public health and primary health care to implement evidence-based strategies could be used to reduce the prevalence of these cancers.
